# Dynamic expression of calretinin in embryonic and early fetal human cortex

**DOI:** 10.3389/fnana.2014.00041

**Published:** 2014-06-03

**Authors:** Miriam González-Gómez, Gundela Meyer

**Affiliations:** Departamento de Anatomía, Facultad de Medicina, Universidad de La LagunaTenerife, Spain

**Keywords:** preplate, pioneer cells, cortical plate, regionalization, lateral migratory stream, ganglionic eminence

## Abstract

Calretinin (CR) is one of the earliest neurochemical markers in human corticogenesis. In embryos from Carnegie stages (CS) 17 to 23, calbindin (CB) and CR stain opposite poles of the incipient cortex suggesting early regionalization: CB marks the neuroepithelium of the medial boundary of the cortex with the choroid plexus (cortical hem). By contrast, CR is confined to the subventricular zone (SVZ) of the lateral and caudal ganglionic eminences at the pallial-subpallial boundary (PSB, or antihem), from where CR+/Tbr1- neurons migrate toward piriform cortex and amygdala as a component of the lateral cortical stream. At CS 19, columns of CR+ cells arise in the rostral cortex, and contribute at CS 20 to the “monolayer” of horizontal Tbr1+/CR+ and GAD+ cells in the preplate. At CS 21, the “pioneer cortical plate” appears as a radial aggregation of CR+/Tbr1+ neurons, which cover the entire future neocortex and extend the first corticofugal axons. CR expression in early human corticogenesis is thus not restricted to interneurons, but is also present in the first excitatory projection neurons of the cortex. At CS 21/22, the cortical plate is established following a lateral to medial gradient, when Tbr1+/CR- neurons settle *within* the pioneer cortical plate, and thus separate superficial and deep pioneer neurons. CR+ pioneer neurons disappear shortly after the formation of the cortical plate. Reelin+ Cajal-Retzius cells begin to express CR around CS21 (7/8 PCW). At CS 21–23, the CR+ SVZ at the PSB is the source of CR+ interneurons migrating into the cortical SVZ. In turn, CB+ interneurons migrate from the subpallium into the intermediate zone following the fibers of the internal capsule. Early CR+ and CB+ interneurons thus have different origins and migratory routes. CR+ cell populations in the embryonic telencephalon take part in a complex sequence of events not analyzed so far in other mammalian species, which may represent a distinctive trait of the initial steps of human corticogenesis.

## INTRODUCTION

The calcium-binding protein calretinin (CR) is a multifunctional protein involved in a variety of activities in the developing and adult brain (Schwaller, 2014). In the adult cortex, CR is mainly expressed in GABAergic interneurons. In most mammalian species, CR+ neurons are concentrated in layers II and III, where they display a bipolar or bitufted, vertically aligned morphology, while multipolar morphologies are observed in deeper layers (Gabbott et al., 1997; Hof et al., 1999; Barinka and Druga, 2010). The double-bouquet cells, an interneuron type characteristic of primate cortex, co-express CR and calbindin (CB; Del Rio and DeFelipe, 1997). In adult human cortex, CR is also present in specific subsets of pyramidal cells, such as the pyramidal-shaped neurons in layers V and VI of entorhinal cortex (Mikkonen et al., 1997), or pyramidal cells in layer V of the anterior cingulate cortex (Hof et al., 2001).

In the rodent, virtually all interneurons have a subpallial origin (reviewed in Welagen and Anderson, 2011). In mice, the majority of CR+ interneurons derive from the caudal ganglionic eminence (CGE; Nery et al., 2002; Xu et al., 2004). These CGE-derived interneurons display bipolar morphologies and may colocalize CR and vasoactive intestinal peptide (VIP; Miyoshi et al., 2010; Lodato et al., 2011). A less common CR+ interneuron subtype with a multipolar morphology co-expresses CR and somatostatin, and may have its origins in the medial ganglionic eminence (MGE; Sousa et al., 2009). The lateral ganglionic eminence (LGE) seems to play a minor role in the generation of interneurons compared to MGE and CGE (see Welagen and Anderson, 2011).

The developmental origins of human interneurons are controversial. For many authors, human and monkey GABAergic interneurons have a double origin, with large proportions deriving from neocortical ventricular and subventricular zones (SVZ), in addition to a lineage originating from ganglionic eminences (GE; Letinic et al., 2002; Meyer et al., 2002b; Rakic and Zecevic, 2003; Petanjek et al., 2009a,b; Jakovcevski et al., 2011; Zecevic et al., 2011; Reinchisi et al., 2012; Al-Jaberi et al., 2013). Recent papers, however, reported that the vast majority of human cortical interneurons are produced in the GE, including a large contribution from non-epithelial SVZ stem cells of the CGE (Hansen et al., 2013; Ma et al., 2013).

The present report is focused on the early period of human corticogenesis, which precedes generation and migration of the large varieties and numbers of interneurons. The human cortex is unique insofar as it is the substrate of our cognitive faculties, and displays an extraordinary structural and functional complexity. It is becoming evident that the mouse, the currently prevalent brain model, is evolutionary too divergent from human to serve as a paradigm for human cortex development. Recent reconstructions of the phenotype of the hypothetical placental ancestor suggested that its brain was gyrencephalic (O’Leary et al., 2013), to the point that the smooth mouse brain may represent a secondary lissencephaly as the result of a phenotypic reversal and a shift toward miniaturization (Kelava et al., 2013). The embryonic and early fetal period sets the framework and pace for the subsequent patterning, proliferation and differentiation events of the cortex. The description and neurochemical definition of its first cellular components are thus crucial for understanding how the human brain is built. Since CR marks early appearing neurons in the human telencephalon (Meyer et al., 2000; Meyer, 2007), co-expression of CR with other proteins may define their possible activities and origins in the initial steps of corticogenesis. In particular, we analyze co-expression of CR and Tbr1, a T-box transcription factor expressed in postmitotic glutamatergic neurons with a pallial origin (Bulfone et al., 1999; Puelles et al., 2000; Hevner et al., 2001). Other proteins co-expressed with CR in the embryonic cortex are Reelin, the extracellular matrix protein secreted by Cajal-Retzius cells (Meyer et al., 1999; reviewed by Tissir and Goffinet, 2003), and GAD (glutamate decarboxylase, the enzyme that catalyzes the decarboxylation of glutamate to GABA). The aim of our analysis is to reconstruct the chain of events that lead to the formation of the cortical plate (CP), the precursor of the adult cortex, in the embryonic human brain.

## MATERIALS AND METHODS

The embryonic and early fetal brains, between 6 and 14 postconceptional weeks (PCW), were the same used in previous studies (Meyer et al., 2000, 2002a,b, 2003). They were obtained after legal abortions following national guidelines in our respective countries, under the supervision of the Ethical Committee of the University La Laguna. The embryos (6, 6.3, 6.5, 7, 7.3, 7.5, 8, 8.5 PCW), were staged according to Carnegie stages (CS) defined by O’Rahilly and Müller (1994). The embryonic heads and fetal brains were fixed in Bouin or 4% paraformaldehyde, embedded in paraffin, and cut in a mostly coronal plane into 7 or 10 μ-thick serial sections. The classification into CS was found more reliable for cortical development than the reported gestational or postconceptional age, where one or two days can result in significant changes of cortical structure. We re-examined previously stained material, in particular immunostaining for CR and CB, and processed unstained sections for Tbr1, Reelin, GAD and PCNA. Double-staining was performed by sequential two-color immunohistochemistry, and confocal microscopy.

### IMMUNOHISTOCHEMISTRY

Sections were deparaffinized, hydrated, and boiled in 10 mM citrate buffer (pH 6) for 20 min for antigen retrieval, rinsed in Tris-buffered saline (TBS, pH 7.6, 0.05 M), and incubated in the primary antibodies overnight in a humid chamber. After rinsing, they were incubated in the corresponding biotinylated secondary antibodies (rabbit anti-mouse IgG or goat anti-rabbit IgG; Dako, Glostrup, Denmark), diluted at 1:200 in TBS, followed by incubation with avidin-biotin complex (ABC, DAKO) in TBS. Bound peroxidase was revealed using 0.04% 3,3-diaminobenzidine (Sigma, USA), 0.05% ammonium nickel (II) sulfate, and 0.03% hydrogen peroxide in TBS, pH 7.6. Sections were dehydrated, cleared, and coverslipped using Eukitt (O. Kindler, Freiburg, Germany). Negative controls omitted the primary antibodies.

### SEQUENTIAL TWO COLOR IMMUNOSTAINING

This method was used because the mouse monoclonal anti-CR antibody gave only faint staining in the younger embryos. Antigens were immunolabeled sequentially by using primary antibodies (Tbr1 and CR) generated in rabbit. The first antibody was developed using DAB/nickel as chromogen. Thereafter, sections were rinsed in TBS and incubated overnight with the second antibody. After incubation with the biotinylated secondary antibodies and ABC as described above, sections were developed by using DAB alone as chromogen. Sections were dehydrated, cleared in xylene, and cover-slipped with Eukitt (Freiburg, Germany). Photographs were taken with a Zeiss Axio microscope equipped with an AxioCamMRc5 digital camera and AxioVision LE 4.6 software. Images were processed using Adobe Photoshop CS2 for adjustment of brightness and contrast.

### DOUBLE IMMUNOFLUORESCENCE

Mouse monoclonal anti- CR or anti-Reelin antibody 142 were mixed with rabbit polyclonal anti-Tbr1 or anti-GAD antibodies and sections were incubated overnight at room temperature. Then the secondary biotinylated anti-mouse IgG antibody (1:400, Amersham) and cyanine-3-coupled anti-rabbit IgG antibody (1:400, Amersham) were incubated for 1 h at room temperature in the dark, followed by cyanine-2 dye conjugated streptavidin (1:400, Amersham) for 1 h. Nuclei were stained with DAPI. Sections were washed in TBS and coverslipped with DABCO (1%) and glycerol-PBS (1:1). Negative controls omitted the primary antibodies. Fluorescence immunosignals were obtained using a Fluoview 1000 laser scanning confocal imaging system (Olympus Optical).

### ANTIBODIES USED

Mouse monoclonal anti-reelin antibody 142 (IgG1, 1:500, de Bergeyck et al., 1998; gift of A. Goffinet),1/500; Mouse monoclonal anti-CR antibody Swant, 6B3, 1/200; Rabbit polyclonal anti-CR, Swant, 7699/4, 1/3000; Mouse monoclonal antibody anti-PCNA, Thermo Scientific, Ab-1 (clone PC10) 1/1000; Rabbit polyclonal anti-CB, Swant, CB-38a, 1/7000; Rabbit polyclonal anti-Tbr1, Abcam, ab31940, 1/300; Rabbit polyclonal antibody anti-GAD 65/67, Abcam, ab49832, 1/1000; Rabbit polyclonal B3 anti-Dab1 antibody, 1/100 (gift of B. Howell).

## RESULTS

### CS17: CR AND CB ARE EXPRESSED AT OPPOSITE BOUNDARIES OF THE CORTEX

**FIGURE 1:**
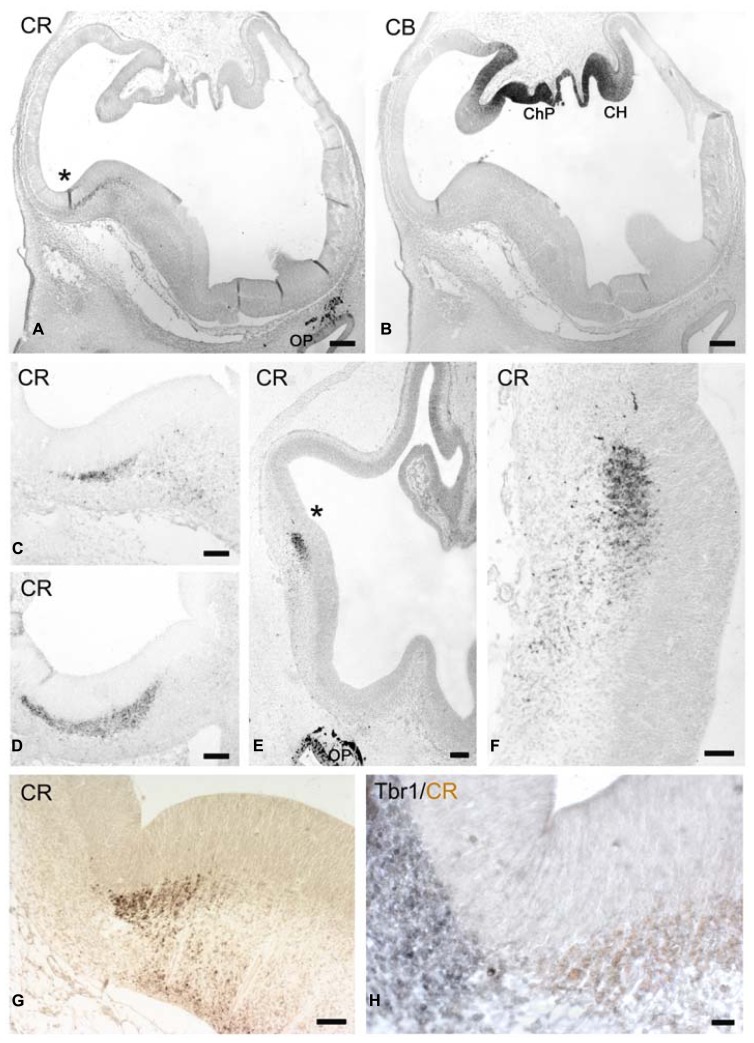
**(A,B)** Low magnification views of human telencephalon at CS17 stained for CR **(A)** and CB **(B)**, which marks the cortical hem (CH). ChP: choroid plexus. **(B) **These coronal sections are slightly oblique, with the right side being more rostral than the left one.** (C)** Shows the CR+ patch in the lateral ganglionic eminence. The cortico-striatal angle is marked by an asterisk in **(A)**. **(D)** Shows the CR+ patch at the level of the caudal ganglionic eminence. **(E–H)** CS18. **(E)** Low magnification view, with the asterisk marking the rostral cortico-striatal angle. **(F)** The CR+ patch in the lateral GE and migration toward the paleocortex. **(G)** A more caudal view of the CR+ patch in the ganglionic eminence, and the ventral migration of the lateral cortical stream. **(H)** Two-color immunostaining of the pallial-subpallial boundary (PSB), with Tbr1 expression (gray) in its cortical aspect, and CR positivity (light brown) in its subpallial sector. Note the intense CR-positivity in the olfactory pit (OP) and its derivatives in **(A,E)**. Scale bars: 100 μm in **(A,B,E)**; 50 μm in **(C,D,F,G,H)**.

In the earliest stage studied here, CS 17 (ca 6 PCW), CR (**Figure 1A**), and CB (**Figure 1B**) were expressed in distinct sectors of the telencephalon. High expression of CB was in the neuroepithelium at the medial edge of the cortical primordium, at the boundary with the choroid plexus anlage, known as the cortical hem, with the developing choroid plexus displaying the strongest CB-intensity (**Figure 1B**). By contrast, CR was present in the dorsal aspect of the LGE near the cortico-striatal angle, or PSB, while the remainder of the GE was CR-negative (we follow the terminology of Pauly et al., 2014). Here, CR-expression was detected in the SVZ along the entire rostro-caudal extent of the GE; few scattered CR+ cells with migratory morphologies were observed in the mantle zone lateral and ventral to the GE, which corresponds to the olfactory paleocortex at rostral levels (**Figure 1C**), and the amygdala at the caudal end (**Figure 1D**). The patch of CR+ SVZ was larger in the CGE (**Figure 1D**). CR positivity was not yet observed in the incipient cortical preplate (at this moment rather a narrow marginal layer), which was occupied only by few small Reelin+/CR- cells representing early born Cajal-Retzius cells just outside the cortical neuroepithelium. Both the cortical hem and the PSB were proposed to be putative signaling centers (Grove et al., 1998; Assimacopoulos et al., 2003; Subramanian et al., 2009), and it is interesting that CB and CR are almost complementary in defining the opposite extremities, hem and antihem, respectively, of the cortical primordium.

### CS 18: CR IN THE LATERAL CORTICAL STREAM

At CS 18 (ca 6.3 PCW; **Figures 1E–H**) the cortico-striatal angle was sharper and thus better defined than at previous stages, and the bulge of the GE was larger and more rounded (compare **Figures 1A,G**, from equivalent levels). Tbr1 marked the lateral, pallial side and CR the medial, subpallial side of the PSB, with a sharp border between them (**Figure 1H**). A SVZ defined by Tbr1+ expression was visible in the cortex lateral to the PBS. The CR+ SVZ of the LGE gave rise to a stream of CR+ neurons that descended toward the olfactory cortex rostrally (**Figures 1E,F**), and to the amygdala caudally, although apparently not dorsally into the cortex (**Figures 1F,G**), forming part of the lateral cortical stream (LCS) of Bayer and Altman (1991, 2006). At even more rostral levels (not shown), CR+ cells migrated ventrally to the retrobulbar area, close to the olfactory bulb anlage. At this moment, the neuroepithelium of the olfactory pit and the olfactory nerve expressed the highest levels of CR (**Figures 1A,E**). The only distinct postmitotic cell population in the early preplate/marginal layer were Reelin+ Cajal-Retzius cells (not shown) which did not yet express CR.

### CS 19: CR AND Tbr1 IN THE INITIAL PREPLATE

**FIGURE 2:**
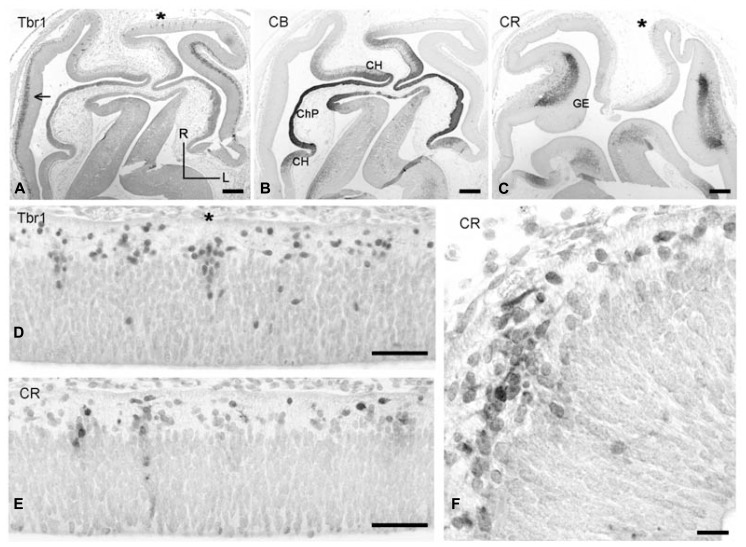
**(A–C)** Low magnification views of horizontal sections through CS 19 brains, stained for Tbr-1 **(A)**, CB **(B),** and CR **(C)**. **(A,B)** Are at a level dorsal to the GE, while **(C)** is at the level of the GE. R, rostral, L, lateral. In **(A)**, the dorsolateral cortex displays a Tbr1+ SVZ (arrow). **(B)** CB defines the choroid plexus anlage (ChP) and adjacent cortical hem (CH). **(C)** CR stains the SVZ of the ganglionic eminences (GE). Fainter CR positivity is also observed in the cortical hem. **(D,E)** Two adjacent dorsal sections showing vertical columns of Tbr1+ **(D)** and CR+ **(E)** in the rostral cortical neuroepithelium. **(F)** Higher magnification of a CR+ column marked in **(C) **with an asterisk, and horizontal migration of CR+ neurons in the preplate. Scale bars: 200 μm in **(A–C)**; 50 μm in **(D,E)**; 25 μm in **(F)**.

Carnegie stages 19 (ca 6.5 PCW) was examined in a particularly well preserved case cut in a horizontal plane (**Figure 2**). This plane allowed us to directly address rostro-caudal differences and reconstruct the three-dimensional organization of the telencephalon at this stage. **Figures 2A,B** shows low magnification views of two dorsal levels of the cortex stained for Tbr1 (A) and CB (B). **Figure 2B** shows the intensely CB+ choroid plexus, and the adjacent hem regions rostrally and caudally to the choroid plexus, which were moderately CB+ and gave rise to a few CB+ cells in the marginal layer, possibly representing hem-derived Cajal-Retzius cells. **Figure 2C** is a more ventral section at the level of the lateral and caudal GEs, the SVZ of which was almost entirely CR+, but Tbr1- (not shown). Examination of sections at levels A and C revealed that the cortical preplate was not homogenous but showed important regional differences. Importantly, a SVZ was observed in A in the lateral cortical sectors adjacent and dorsal to the GEs (which would be below this level, and not be visible in this horizontal plane of section). The cortical SVZ of the embryonic stages was Tbr1+ (**Figure 2A**; see also Bayatti et al., 2008b) and CR- (not shown). In turn, the cortical sectors rostral and caudal to the GE did not yet have a SVZ. However, the rostral sector (possibly representing the future prefrontal areas) displayed focal columns of Tbr1+ cells which exited the VZ and streamed into the marginal layer, or incipient preplate, where they adopted a horizontal orientation (**Figure 2D**). Similar columns of apparently postmitotic cells in the same location were CR+ (**Figures 2E,F**), suggesting colocalization of Tbr1 and CR in postmitotic cells in the rostral cortex.

The cortical sectors caudal to the GE (probably future temporal and occipital areas) did not show focal aggregations of Tbr1+ or CR+ cells, but only a homogeneous proliferative VZ and a narrow marginal layer containing few Reelin+ Cajal-Retzius cells.

### CS 20: CR AND Tbr1 IN THE ADVANCED PREPLATE

**FIGURE 3:**
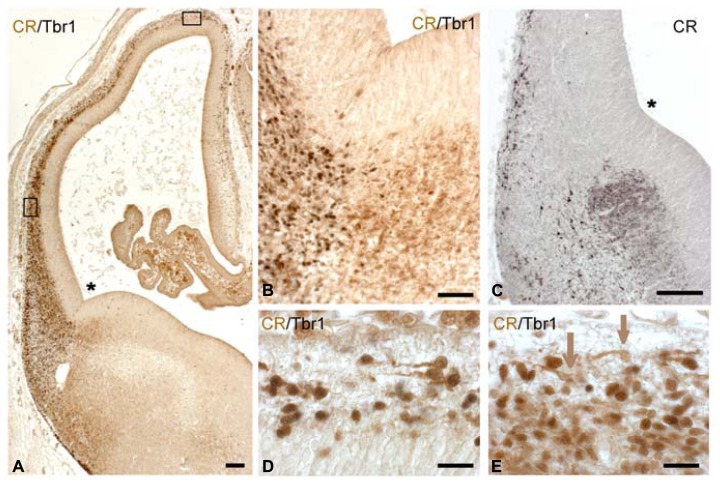
**CS 20. (A)** Low magnification view of a coronal section doubly stained for Tbr-1 (dark brown or gray) and CR (light brown). **(B)** The PSB at higher magnification, showing a clear demarcation between the pallial, Tbr1+ side and the Tbr1-negative, CR+ subpallial side. **(C)** The CR+ patch in the LGE gives rise to a lateral and ventral migration stream; the monolayer of CR+ cells appears in the preplate. **(D,E)** Two-color immunohistochemistry of the dorsal **(D)** and lateral sector **(E)**, indicated by boxes in **(A)**. Dorsally, Tbr1+/CR+ cells predominate (dark nuclear staining with Tbr1), while more ventrally, both Tbr1-/CR+ (brown arrows) are side by side with Tbr1+/CR+ horizontal cells. The SVZ is lightly stained for Tbr1. Asterisks mark the cortico-striatal angle. Scale bars: 100 μm in **(A,C)**; 50 μm in **(B)**; 25 μm in **(D,E)**.

At CS 20 (ca 7 PCW) further complexity was observed in the cell composition of the developing cortex, which presents a more advanced preplate (**Figure 3**). A Tbr1+ SVZ was now recognizable throughout almost the entire cortex, although it was thickest near the PSB adjacent to the LGE (**Figure 3A**). The PSB was clearly delimited by Tbr1-positivity on the pallial side, and CR-positivity on the subpallial side (**Figures 3A,B**). Double immunofluorescence for PCNA and CR (not shown) did not provide evidence for co-expression of both markers and suggested that the CR+ cells in the LGE were not proliferating. The prominence of the SVZ was in parallel with an increasing cellularity of the preplate; both followed a gradient decreasing from lateral to medial, as well as decreasing gradients toward the rostral and caudal poles of the cortex. The preplate overlying the SVZ was dominated by horizontal bipolar or monopolar CR+ cells (**Figures 3D,E**; the monolayer, Meyer et al., 2000). Even though the CR+ cells displayed a morphology usually attributed to Cajal-Retzius cells, they were Reelin-; the Reelin+ Cajal-Retzius cells at this stage were located closer to the pial surface and not yet CR+. We reported previously that some horizontal monolayer-cells expressed GAD (Meyer et al., 2000). We now analyzed the co-expression of CR and Tbr1 in the CS 20 preplate, and found that Tbr1+/CR+ cells predominated dorsally and medially (**Figure 3D**), whereas Tbr1-/CR+ cells were intermixed with Tbr1+/CR+ neurons in lateral regions (**Figure 3E**). The Tbr1- cells probably correspond to the GAD+/GABA+ cells previously described at this stage (Zecevic and Milosevic, 1997; Meyer et al., 2000; Rakic and Zecevic, 2003; Zecevic et al., 2011). Most of the CR+ cells migrating away from the LGE (**Figure 3C**) appeared to take a ventral and lateral route toward paleocortex and lateral neocortex following the LCS (Bayer and Altman, 1991) rather than traversing the PSB in a dorsal direction. An open question was the origin of the Tbr1+/CR+ cells in the advanced preplate, since cells in the cortical SVZ were Tbr1+ but did not express CR. We suggest that a possible origin of the Tbr1+/CR+ monolayer cells may be the Tbr1+/CR+ columns observed in the rostral cortical sector at CS 19, which would point to long-range tangential migrations within the preplate.

### CS 21: THE EMERGENCE OF THE PIONEER CORTICAL PLATE

**FIGURE 4:**
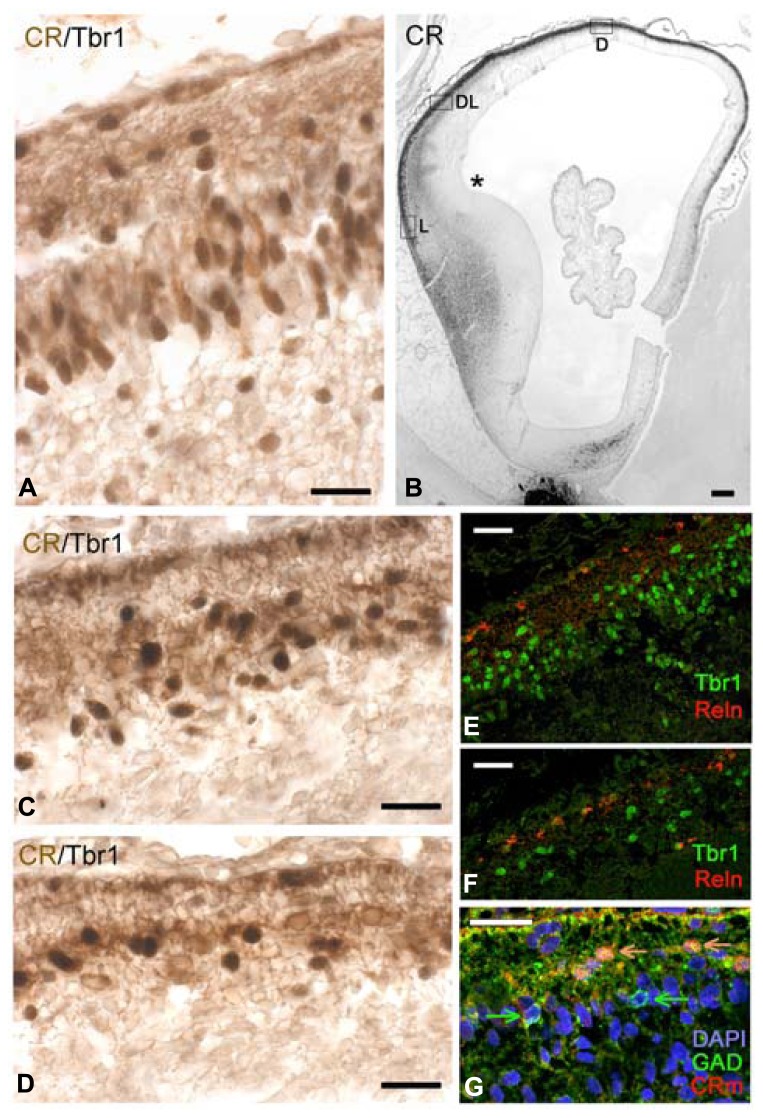
**CS 21. (A)** Corresponds to a lateral (L), **(C)** to a dorsolateral (DL), and **(D) **to a dorsal (D) sector of the low magnification view in **(B)**, indicated by boxes. **(B)** Is immunostained for CR; **(A,C,D)** are doubly stained for Tbr1 (dark nuclei) and CR (light brown cytoplasm). Note that the lateral sector in **(A)** already shows a radially oriented pioneer cortical plate, whereas in **(C)**, Tbr1+/CR+ cells are intermixed with Tbr1-/CR+ cells, without any radial orientation. **(D)** Corresponds to a monolayer stage, with most cells showing a horizontal orientation, and Tbr1+/CR+ cells being outnumbered by Tbr1-/CR+ cells. **(E,F)** Confocal microscopy of Reelin+ Cajal-Retzius cells in a subpial location, segregated from pioneer plate **(E)** and monolayer cells **(F)**, marked with Tbr1. The level of **(E)** corresponds to that in **(A)**, the level of **(F)** to that of **(D)**. **(G)** Confocal microscopy of a dorsal sector of this case, showing colocalization of GAD and CR in superficial, horizontally oriented cells (orange arrows), while GAD alone was expressed in a deeper population (green arrows). Note that the mouse monoclonal CR-antibody was less sensitive than the rabbit CR antibody routinely used in the other panels. The asterisk in B marks the cortico-striatal angle. Scale bars: 100 μm in **(B)**; 50 μm in **(E,F)**; 25 μm in **(A,C,D,G)**.

Carnegie stages 21 (ca 7.5 PCW) marks a turning point in corticogenesis: the condensation of the loosely arranged preplate into a compact cell “plate,” the pioneer CP, or “pioneer plate” of Meyer et al. (2000). This aggregation of radially oriented neurons appeared first near the PSB (**Figure 4A**), while more dorsally we still observed a stage resembling the monolayer (**Figure 4D**), with a transitional, non-radially oriented compact cell condensation in a dorso-lateral position (**Figure 4C**). The lateral to medial progression of the pioneer CP varied among the fetuses of similar ages examined (7–8 PCW).

The most advanced pioneer CP with a thickness of several cell layers was found close to the future entrance of the internal capsule which at this stage was visible below the GE as a compact CB+ fiber bundle (not shown), but had not yet entered the cortex. Rostral and caudal cortical sectors lagged slightly behind. Virtually all cells of the pioneer CP were intensely CR+, and gave rise to a loose CR+ axonal plexus that followed a ventral and lateral course, representing the first corticofugal fibers (best visible in **Figure 4B**).

The apparently homogeneous CR+ pioneer CP of CS 21 turned out to be rather heterogeneous. Two-color immunohistochemistry (confocal microscopy using the monoclonal mouse CR antibody often failed in the early stages) revealed that both Tbr1+ and Tbr1- neurons formed part of the CR+ pioneer CP, as well as of the transitional dorsolateral cell condensation and the dorsomedial monolayer (**Figures 4A,C,D**). In the dorsolateral (**Figure 4C**) and dorsomedial (**Figure 4D**) regions,

Tbr1-/CR+ cells even seemed to outnumber Tbr1+/CR+ neurons. This finding may be interpreted as a massive and rather fast invasion of the preplate by Tbr1- cells, probably GABAergic neurons, compared to the rather slow emergence of the Tbr1+ SVZ which seemed to lag behind (compare the numbers of Tbr1+ cells in the dorsal cortex, compared to the CR+/Tbr1- cells). Furthermore, there was evidence of GAD+/CR+ and GAD+/CR- cells, as well as for Tbr1+/CR- cells. The GAD+/CR+ cells seemed to occupy a more superficial position, whereas the GAD+/CR- cells lay deeper (**Figure 4G**). The complexity of the advanced preplate cell populations points to an important role of GABAergic neurons in the earliest stages of cortex formation. Cajal-Retzius cells, characterized by Reelin-expression and a subpial location (**Figures 4E,F**) now also began to express CR, or CB when they were close to the cortical hem.

### CS 22/23: THE TRANSITION FROM PIONEER PLATE TO CORTICAL PLATE

**FIGURE 5:**
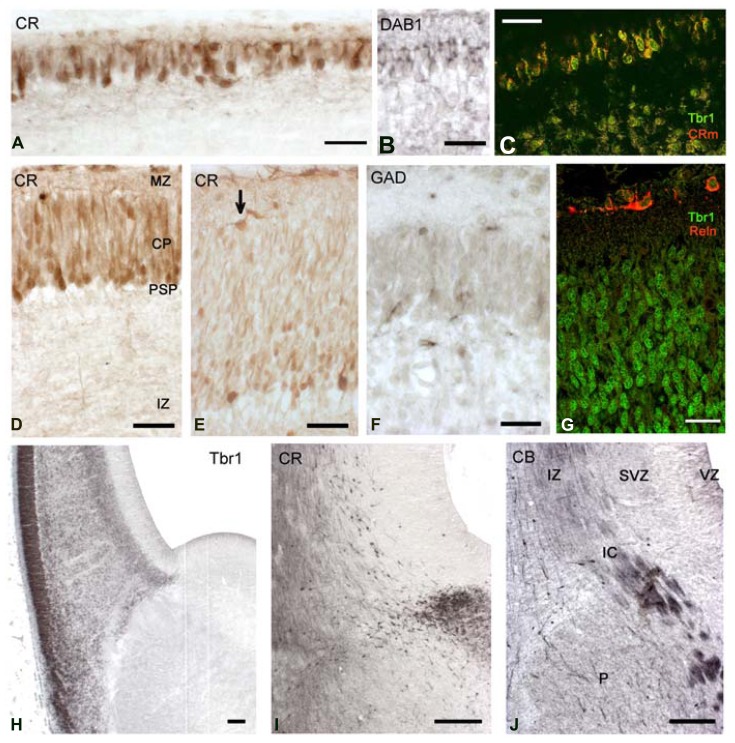
**CS22/23.** Coronal sections from an 8.5 PCW-old case presenting pioneer cortical plate, cortical plate and intermediate stages. **(A–C)** The pioneer cortical plate, in the dorsal and medial sector of the cortex, is positive for CR **(A)**, Dab1 **(B)** and both Tbr1 and CR **(C)**, using confocal microscopy and the mouse monoclonal CR antibody (CRm). The pioneer cortical plate has a deep component of pyramidal-like neurons, and a superficial component of more rounded cells **(A)**. Both give rise to an axonal plexus beneath the pioneer plate **(A)**, and express Dab1 in the apical tips of their cytoplasm **(B)**. Pioneer cells co-express Tbr1 and CR **(C)**.** (D)** At an intermediate level, the cortical plate (CP) appears as lightly CR+ cells, while few superficial pioneer cells are still evident, and deep pioneer cells form the presubplate (PSP). **(E)** In the lateral sector, the cortical plate is widest, and CR-positivity has largely disappeared. Superficial pioneer cells are rare (arrow). **(F)** After the emergence of the CP, GAD+ cells are rare and mostly below and above the CP. **(G)** The CP is composed of Tbr1+ cells, and Reelin+ Cajal-Retzius cells appear now more differentiated. **(H)** Low magnification view of the PSB stained for Tbr1. Note the migration toward the ventral and lateral cortical areas, forming the lateral cortical stream, and the sharp delineation of the subpallium. **(I)** The CR+ patch in the LGE gives now rise to a dorsal migration stream into the SVZ of the cortex. Compare with **(J)** The first CB+ fibers of the internal capsule (IC) enter the cortex through the intermediate zone (IZ). CB+ interneurons migrate with the fibers, taking a more superficial route than the CR+ cells. P, Putamen. Scale bars: 100 μm in **(H–J)**; 50 μm in **(A–G)**.

The CP emerged at CS 22/23 (ca 8 PCW). There was actually a gradual transition from the radially organized pioneer CP, still visible in dorsal and medial areas (**Figures 5A–C**), and the CP in lateral, more advanced areas (**Figures 5D,E**). Pioneer cells were large neurons, particularly the deeper ones which had pyramidal-like shapes and may represent the future presubplate (Meyer et al., 2000; reviewed by Kostovic et al., 2011), while the superficial ones were more rounded (**Figure 5A**) and became increasingly sparse as the CP progressed. The axonal plexus had its origin in superficial and deep pioneer cells (see Meyer et al., 2000). The pioneer CP is an important step in cortex formation, because for the first time we observed a distinct expression of Dab1 in the apical tips of pioneer cells (**Figure 5B**), a staining pattern characteristic of the CP. Dab1 is a crucial component of the Reelin-Dab1 signaling pathway that responds to Reelin secreted by Cajal-Retzius cells (Meyer et al., 2003, reviewed by Tissir and Goffinet, 2003). Confocal microscopy revealed that pioneer neurons co-expressed Tbr1 and CR (**Figure 5C**), which is another feature of pyramidal neurons, demonstrating that they were indeed excitatory projection neurons. The CP was formed through the continuous arrival of new Tbr1+ cells and their insertion within the framework provided by the superficial and deep pioneer cells. CP-cells were lightly stained for CR in the dorso-lateral cortex (**Figure 5D**), and CR- in the lateral cortex (**Figure 5E**). In the latter, CR-positivity was still prominent in the presubplate and in the few remnants of the superficial pioneer cells. GAD-immunoreactivity was restricted to interneurons preferentially located along the upper and lower borders of the CP (**Figure 5F**).

Carnegie stages 22 and 23 were difficult to separate, because the five cases in our 8–10 PCW group were quite similar, and differed mainly in the size of the hemisphere, with all cases presenting a pioneer CP medially, and a multilayered CP laterally. Of note, this age represented another remarkable step in corticogenesis: the arrival of the internal capsule in the cortex, after traversing the PSB. The internal capsule was initially CB+ and accompanied by CB+ interneurons streaming into the intermediate zone (**Figure 5J**). The CR+ patch in the LGE adjacent to the PSB was now also the origin of a stream of CR+ interneurons which migrated into the SVZ, taking a deeper route than the CB+ interneurons (compare **Figures 5I,J**).

The pallial side of the PSB was clearly delineated by Tbr1 expression (**Figure 5H**); Tbr1 positivity was highest in the CP, but also visible in the SVZ and in neurons migrating radially through the IZ. Tbr1 also marked the LCS contributing pyramidal cells for paleocortex and insula. Cajal-Retzius cells, defined by Reelin-expression, had a more differentiated morphology (**Figure 5G**) and now clearly co-expressed CR (**Figures 5D,E**).

### THE EARLY FETAL STAGE: DIFFERENTIATION OF INSULA AND TEMPORAL LOBE

**FIGURE 6:**
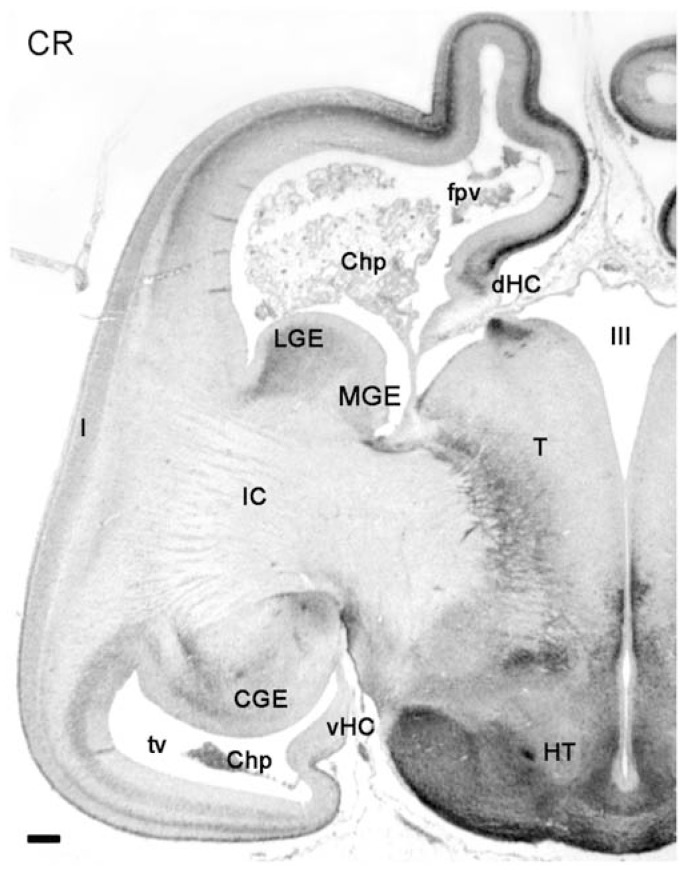
**Low magnification view of a hemisphere at 11PCW, stained for CR.** The early fetal stages are characterized by growth and differentiation processes which, among many other features, allow the distinction of the temporal horn of the lateral ventricle (tv), the enormous growth increase of the internal capsule (IC) and the insula (I), the ventral position of temporal lobe and caudal ganglionic eminence (CGE), and the appearance of the ventral hippocampus (vHC). The intensely CR+ pioneer cortical plate is still recognizable in the frontal and parietal lobes, but not evident in the temporal lobe. CR is expressed in the LGE and the CGE. Other abbreviations: Chp, choroid plexus; fpv, fronto-parietal ventricle; dHC, dorsal hippocampus; HT, hypothalamus; MGE, medial ganglionic eminence; T, thalamus; III, third ventricle. Scale bar: 100 m.

The focus of this work is on the embryonic stages of corticogenesis rather than on the fetal cortex. However, as an indication of the ongoing complexity of human cortex development we show in **Figure 6** a coronal section of an 11-PCW old fetus immunostained for CR to point out a few important differences with rodent cortex that become evident after the embryonic period: first, the formation of a temporal lobe ventral to a largely increased insula; second, the ventral position of the CGE protruding into the temporal horn of the lateral ventricle; third, the mirror-like arrangement of the PSB in the fronto-parietal cortex dorsally, and the temporal cortex ventrally, with both displaying a CR+ patch in the lateral aspect of the GEs. It is clear from this figure that the medial temporal lobe does not show a CR+ pioneer CP, and thus apparently does not follow the sequence of events which we described for the fronto-parietal cortex, and which applies also to the prefrontal and occipital areas. Many aspects of the human cortex are as yet unknown, and will need a continued effort and much further work to understand its singularity.

## DISCUSSION

Calretinin is expressed in early appearing neurons in the human embryonic and early fetal cortex, and analysis of CR immunostaining – along with that of other marker molecules – allowed us to obtain insight into early occurring telencephalic regionalization, and to distinguish a sequence of events that lead from the incipient preplate to the formation of the CP.

### EXPRESSION OF CR AND CB REFLECTS AN EARLY REGIONALIZATION OF THE HUMAN CORTEX

Already in the earliest stage examined, CS 17 (ca 6 PCW), CR is defining the lateral domain of the GE, which directly abuts the ventral border of the future cortex. The PSB is delineated by the expression of Tbr1, a marker of pallial glutamatergic cells (Bulfone et al., 1999; Puelles et al., 2000; Hevner et al., 2001), in the lateral aspect of the cortico-striatal angle, and the presence of CR in cells medial to this angle, with the absence of Tbr1 indicating their subpallial GABAergic identity. We report here that CR+ cells in the SVZ of the LGE are among the first postmitotic cells of the telencephalon, and differentiate earlier than cells in the pallium, with the exception of the first cohort of Cajal-Retzius cells (Meyer et al., 2000). What we find interesting is the positivity for CB in the medial aspect of the hemisphere, where cortical hem and choroid plexus are developing. CB is an early marker of the human hem system (Meyer, 2010; Roy et al., 2013), which includes the medial edge of the cortex as a putative signaling center involved in patterning of hippocampus and cortex (Grove et al., 1998), as well as the boundaries of prosencephalic structures such as septum and thalamic eminence with the prospective choroid plexus (Meyer, 2010; Roy et al., 2013). The structures belonging to the hem system express unique complements of signaling molecules, and require the transcription factor Lhx2 to delimit their extent (Roy et al., 2013). The PSB was proposed to represent an antihem, a putative signaling center at the opposite, lateral end of the pallium (Assimacopoulos et al., 2003). While the hem is characterized by the expression of Wnt and Bmp-genes (Grove et al., 1998), the antihem expresses the Wnt antagonist Sfrp2, members of the epidermal growth factor family, and Fgf7 (Assimacopoulos et al., 2003; Subramanian et al., 2009). It is tempting to propose that CR defines cell populations of the antihem, and CB of the hem, even though these calcium-binding proteins may not be directly involved in the signaling cascades. The first derivatives of the hem are CB+/p73+ Cajal-Retzius cells (Meyer et al., 2002a), whereas the first postmitotic neurons in the antihem form the CR+ cell cluster in the LGE that will be discussed below. In the mouse, neither CB nor CR are present in hem and antihem, which can be detected only through the expression of their characteristic morphogens and transcription factors. The coordinated activity of hem and antihem may be involved in delimiting the extent of the proliferative neuroepithelium of the future cortex.

In addition, and possibly independently from the hem/antihem signaling centers, the distinct and rather selective columnar expression pattern of CR/Tbr1 in the prospective frontal lobe at CS 19 suggests a role of CR/Tbr1 in cortical patterning, since Tbr1 is a transcription factor involved in promoting frontal identities in postmitotic neurons and in modulating the balance of cortical arealization (Bedogni et al., 2010). We want to point out the difference of CR expression in the subpallium, where it marks GABAergic interneurons, and CR expression in the pallium, where it can be associated with Tbr1, a marker of glutamatergic projection neurons. CR expression is thus *a priori* unrelated to the GABAergic or glutamatergic signature of a neuron.

### CR MARKS AN EARLY MIGRATION FROM THE PSB VIA THE LATERAL CORTICAL STREAM

The CR+ domain in the SVZ of LGE and CGE appeared already at CS17, along with the onset of a migration of initially few CR+ cells toward the ventral areas, which were, from rostral to caudal, the retrobulbar area, future piriform cortex and amygdala. This migration became more prominent in the following stages when the morphology and architecture of the ventral telencephalon increased in complexity. The complementary expression of Tbr1 and CR in the PSB points to a parallel migration from both sides of the cortico-striatal border in a lateral and ventral direction and indicates that both form part of the LCS described by Bayer and Altman (1991) in the rat and identified also in human (Bayer and Altman, 2006). The LCS, also described as the lateral and ventral migratory streams (Medina et al., 2004), has been studied extensively in the rodent. According to Bayer and Altman (1991), the LCS contributes neurons to various ventral forebrain structures including piriform cortex and amygdala. Further destinations are the lateral neocortex, claustrum, endopiriform nucleus and olfactory tubercle, or, more generally, centers belonging to the lateral and ventral pallium of chick and mice (Puelles et al., 2000; Tamamaki et al., 2001; Medina et al., 2004; Bai et al., 2008). The LCS is formed by heterogeneous cell populations arising from distinct compartments of the PSB; transcription factors Tbr1, Pax6, and Emx2 are expressed in cells emerging from the pallial compartment, whereas cells arising in the subpallial compartment express Dlx2 or co-express Pax6 and Dlx2 (Puelles et al., 2000; Carney et al., 2006). In addition, a specific histogenetic zone at the PSB is defined by Dbx1, which provides cells for the ventral pallium (Puelles et al., 2000; Medina et al., 2004), and for an early born transient glutamatergic cell population migrating over the entire CP, plus a subset of Cajal-Retzius cells (Teissier et al., 2010) which we could not confirm in human.

The PSB described here in human embryos may have played a crucial role in the evolution of the mammalian forebrain through the LCS (Molnár and Butler, 2002). We want to point out some features that may be relevant for human cortical development. Importantly, the cell populations (Tbr1+ and CR+) on their respective sides of the PSB arise very early in embryonic life, at CS 17 and perhaps even earlier, marking the onset of cortical development. Tbr1+ glutamatergic neurons populate the olfactory paleocortex, amygdale, and future lateral neocortex concurrently with Tbr1-/CR+ putative GABAergic neurons via the LCS. However, as shown in **Figure 6**, shortly after the embryonic period, areal patterning and architecture of the lateral and ventral neocortical areas take a distinct course in human development, which is not comparable to that of rodents. This is to say that the PBS and LCS of the human embryo implicitly contain the progenitor areas and future migratory pathways necessary for the formation of the insular lobe in parallel with an expansion of the claustrum, and the establishment of the temporal lobe ventral to the insula. Interestingly, 3-D models of the telencephalon of CS19 human embryos indicated a relative size increase of the region corresponding to the ventral pallium of mice, which was interpreted as a sign of higher complexity of the human claustroamygdaloid structures (Lindsay et al., 2005). The term “ventral pallium” (Puelles et al., 2000) has different connotations in mouse and human, and much further work is needed to fill in the gap between human and rodent-related knowledge.

The origin of interneurons from transcription factor Dlx1/2 and Nkx2.1 domains in the GE is a well-established fact in rodents (reviewed by Welagen and Anderson, 2011). In primates, the situation is more complex and in part disputed. Our finding of an early CR+ domain in the LGE and CGE that gives rise first to a latero-ventral migration, and later, at CS 22, to a dorsal migration into the cortex, does not imply that during fetal life all CR+ interneurons derive from LGE and CGE. Fetal interneuronogenesis is complicated by the fact that significant numbers of GABAergic cells emerge from the cortical VZ and SVZ (Letinic et al., 2002; Rakic and Zecevic, 2003; Petanjek et al., 2009a,b; Zecevic et al., 2011; Jakovcevski et al., 2011; Reinchisi et al., 2012; Al-Jaberi et al., 2013). The LGE and CGE may be the origins only of the first of successive CR+ interneuron migrations, most of which occur later in development and in different places.

### CALRETININ AND Tbr1 DEFINE DISTINCT CELL POPULATIONS IN THE HUMAN PREPLATE

The original concept of the preplate, or primordial plexiform layer, postulated that its components – early born Cajal-Retzius cells and subplate cells – are split by the arrival of later born cortical-plate cells (Marin-Padilla, 1978). This concept, based on the Golgi method, was modified and adapted to the human (Meyer et al., 2000), primate (Smart et al., 2002) and rodent (Meyer et al., 1998) cortex with the advent of immunohistochemistry and the appearance of neurochemical markers for specific cell types.

In human embryos, the preplate is continuously changing because newly arriving cell components are added at different moments of development (Zecevic and Milosevic, 1997; Meyer et al., 2000, 2002b; Rakic and Zecevic, 2003; Zecevic et al., 2011). Initially, from 5.5 to 6.3 PCW (CS 16–18), the early marginal layer of the prospective neocortex contains only Reelin+ Cajal-Retzius cells that may have originated locally from Reelin+ radial cell columns in the neuroepithelium (Meyer et al., 2000, 2002b), shortly before the cortical hem produces additional Reelin+/p73+ Cajal-Retzius cells (Meyer et al., 2002a). In agreement with our previous work, we consider CS 19 (6.5 PCW) as the initial preplate stage, since for the first time non-Cajal-Retzius cells appear in the future neocortex. We show now that CR+ cells at CS 19 originate from focal columns embedded in the rostral cortical neuroepithelium, similar to Tbr1+ cells, and suggest that Tbr1+/CR+ cells migrate tangentially through the preplate and form part of the “monolayer” of horizontal cells characteristic of the advanced preplate at CS 20 (Meyer et al., 2000). The next step would be the aggregation of Tbr1+/CR+ cells in the compact pioneer CP at CS 21, and the appearance of the first pioneer axons of the neocortex.

The neurochemical heterogeneity of the advanced preplate complicates a straightforward interpretation of these early events. We show that at CS 20 (ca 7 PCW) and CS21(ca 7.5 PCW), Tbr1+/CR+ cells coexist with Tbr1-/CR+ cells, with the latter probably representing part of the GABAergic neurons which are a prominent part of the advanced preplate. We did not observe local aggregations or columns of GAD+ cells in cortical territory at earlier stages, and rather propose a subpallial origin for Tbr1- preplate cells. In fact, Rakic and Zecevic (2003) provided evidence that a subset of GABAergic cells in the CS 19 and 20 preplate co-expressed either Nkx2.1 or Dlx transcription factors, which is consistent with their origin in GE and subsequent tangential migration into the preplate (Zecevic et al., 2011). Alternatively, or in addition, GABAergic cells may also be born locally in distinct sectors of the cortical neuroepithelium (Rakic and Zecevic, 2003; Al-Jaberi et al., 2013). The prominence of GABAergic cells in the advanced preplate is supported by the work of Ben-Ari (reviewed by Ben-Ari et al., 2004) who described that GABAergic neurons act as the true pioneers in the developing cortical network, establishing functional synapses earlier than glutamatergic projection neurons. According to this author, the early established GABAergic synapses are depolarizing, and may modulate the maturation of immature, silent principal neurons. The time point of the massive presence of GABA in the monolayer is just a few days/hours before the emergence of the pioneer plate and the emission of the first projection fibers from pioneer neurons. In fact, the coexistence of pioneer CP and monolayer in the lateral and medial regions, respectively, in the same embryo suggests that in a very short time the cells of the monolayer aggregate to form the compact pioneer CP.

### CR AND Tbr1 IN PROJECTION NEURONS OF PIONEER CORTICAL PLATE AND CORTICAL PLATE

The human pioneer CP is a short-lived and transient structure, which is important because it constitutes the first radially organized, Tbr1+ cell layer that gives rise to the efferent pioneer projections. The transitional stages between pioneer CP and CP, which follow the general maturation gradient from lateral to medial (Bayer and Altman, 1991), indicate that preplate splitting consists actually of a splitting of the pioneer CP, when CR- CPcells settle between the superficial and deep CR+ pioneer cells. CR in pioneer cells should not be interpreted as a marker of interneuron identity, since CR and Tbr1 colocalize in the pioneer CP. The putatively glutamatergic pioneer cells may derive from the CR+ radial cell columns observed at CS 19 in the rostral cortex, which may adopt a bipolar migratory morphology at CS 20, and assemble in the compact pioneer plate at CS 21. CR expression in these early cell populations may be related to specific functional roles of this calcium-binding protein which are as yet unknown, but probably independent of neurotransmitter identity.

The pioneer CP is not restricted to human cortex. In the rat, pioneer cells at embryonic day 13 express CB and CR (Meyer et al., 1998), although they do not in the mouse (Espinosa et al., 2009). The rodent pioneer neurons emit the first fiber bundle of the cortex which directs toward the GE and the internal capsule but probably does not cross the telencephalic-diencephalic boundary (Meyer et al., 1998; Espinosa et al., 2009). The deep pioneer neurons are usually identified as subplate cells, while the presence of superficial pioneer cells in MZ and superficial CP is easily overlooked. In fact, human superficial pioneer cells are rare in the lateral CP at CS 23 (ca 8.5 PCW), which may be due to cell death or dilution in a rapidly growing cortex. We do not want to enter here the discussion on the human subplate, which has been the subject of many comprehensive studies and reviews demonstrating that the deep pioneer neurons rather represent a presubplate (Meyer et al., 2000; Smart et al., 2002; Meyer, 2007; Kostovic et al., 2011). The human subplate arises around 10 PCW, expands after 13 PCW (Bayatti et al., 2008a), attains highest differentiation in the second half of gestation, and may not be directly comparable to the rodent subplate.

We pointed out previously (Meyer et al., 2000) that Cajal-Retzius cells do not form part physically of the splitting of the preplate, because already at CS 19 they are confined to a subpial position, spatially segregated from the other cell populations in the preplate. However, the Reelin signal in Cajal-Retzius cells is crucial for preplate (or rather pioneer CP) splitting, because absence of Reelin prevents this process, leading in mice to the *reeler* phenotype characterized by a roughly inverted cortex (reviewed by Tissir and Goffinet, 2003). The Dab1+ pioneer plate may represent the first target population of Reelin-Dab1 signaling activity involved in radial migration.

As discussed here, the embryonic development of the human cortex leaves open many questions that require further clarification. In particular, the possible birthplace of early GABAergic neurons and Tbr1+ pioneer neurons in specific sectors of the cortical neuroepithelium, followed by tangential pathways through the preplate, requires further investigation. The difficulties and ethical limitations in obtaining well preserved human embryos make it imperative to explore other animal models, beyond the prevailing but insufficient mouse model.

## Conflict of Interest Statement

The authors declare that the research was conducted in the absence of any commercial or financial relationships that could be construed as a potential conflict of interest.
